# [2,7-Dimeth­oxy-8-(2,4,6-trimethyl­benzo­yl)naphthalen-1-yl](2,4,6-trimethyl­phen­yl)methanone

**DOI:** 10.1107/S1600536811051579

**Published:** 2011-12-03

**Authors:** Toyokazu Muto, Kosuke Sasagawa, Akiko Okamoto, Hideaki Oike, Noriyuki Yonezawa

**Affiliations:** a Department of Organic and Polymer Materials Chemistry, Tokyo University of Agriculture & Technology, 2-24-16 Naka-machi, Koganei, Tokyo 184-8588, Japan

## Abstract

In the title compound, C_32_H_32_O_4_, the dihedral angle between the two benzene rings of the 2,4,6-trimethyl­benzoyl groups is 71.43 (7)°. The dihedral angles between the two benzene rings and the naphthalene ring system are 81.58 (5) and 84.92 (6)°. An intra­molecular C—H⋯O inter­action is observed.

## Related literature

For electrophilic aromatic substitution of naphthalene deriv­atives, see: Okamoto & Yonezawa (2009 ▶); Okamoto *et al.* (2011 ▶). For the structures of closely related compounds, see: Muto *et al.* (2010 ▶, 2011*a* ▶,*b* ▶).
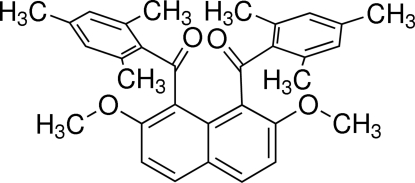


## Experimental

### 

#### Crystal data

C_32_H_32_O_4_
*M* *_r_* = 480.58
Monoclinic, 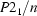

*a* = 7.71685 (14) Å
*b* = 29.2344 (5) Å
*c* = 11.5567 (2) Å
β = 102.879 (1)°
*V* = 2541.57 (8) Å^3^
*Z* = 4
Cu *K*α radiation
μ = 0.65 mm^−1^
*T* = 193 K
0.60 × 0.40 × 0.10 mm

#### Data collection

Rigaku R-AXIS RAPID diffractometer
Absorption correction: numerical (*NUMABS*; Higashi, 1999 ▶) *T*_min_ = 0.697, *T*_max_ = 0.938
45450 measured reflections
4657 independent reflections
4131 reflections with *I* > 2σ(*I*)
*R*_int_ = 0.054

#### Refinement

*R*[*F* ^2^ > 2σ(*F* ^2^)] = 0.041
*wR*(*F* ^2^) = 0.121
*S* = 1.09
4657 reflections
334 parameters
H-atom parameters constrained
Δρ_max_ = 0.26 e Å^−3^
Δρ_min_ = −0.26 e Å^−3^

### 

Data collection: *PROCESS-AUTO* (Rigaku, 1998 ▶); cell refinement: *PROCESS-AUTO*; data reduction: *CrystalStructure* (Rigaku/MSC, 2004 ▶); program(s) used to solve structure: *SIR2004* (Burla *et al.*, 2005 ▶); program(s) used to refine structure: *SHELXL97* (Sheldrick, 2008 ▶); molecular graphics: *ORTEPIII* (Burnett & Johnson, 1996 ▶); software used to prepare material for publication: *SHELXL97*.

## Supplementary Material

Crystal structure: contains datablock(s) I, global. DOI: 10.1107/S1600536811051579/vm2138sup1.cif

Structure factors: contains datablock(s) I. DOI: 10.1107/S1600536811051579/vm2138Isup2.hkl

Supplementary material file. DOI: 10.1107/S1600536811051579/vm2138Isup3.cml

Additional supplementary materials:  crystallographic information; 3D view; checkCIF report

## Figures and Tables

**Table 1:** Hydrogen-bond geometry (Å, °)

*D*—H⋯*A*	*D*—H	H⋯*A*	*D*⋯*A*	*D*—H⋯*A*
C29—H29*C*⋯O2	0.98	2.33	3.1669 (19)	143

**Fig. 1.**
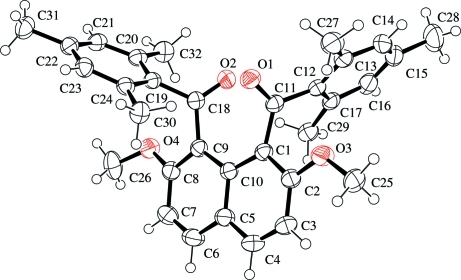
The molecular structure of the title compound with displacement ellipsoids drawn at the 50% probability level.

**Fig. 2.**
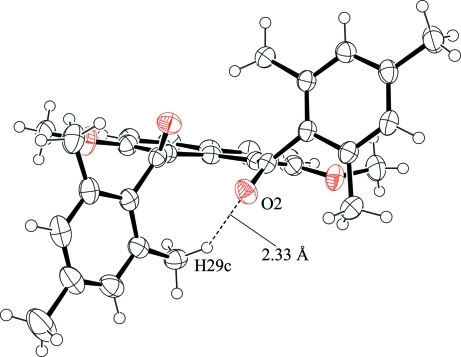
Intramolecular C—H···O interaction shown as dashed line.

## supplementary crystallographic information

### Comment

In the course of our study on the electrophilic aromatic aroylation of 2,7-dimethoxynaphthalene, *peri*-aroylnaphthalene compounds have proven to be formed regioselectively with the aid of suitable acidic mediators (Okamoto & Yonezawa, 2009; Okamoto *et al.*, 2011). Recently, we have reported the crystal structures of several 1,8-diaroylated naphthalene analogues exemplified by 1,8-bis(4-methylbenzoyl)-2,7-dimethoxynaphthalene (Muto *et al.*, 2010). The aroyl groups at the 1,8-positions of the naphthalene rings in these compounds are connected in an almost perpendicular fashion. Besides, the crystal structures of 1-monoaroylated naphthalene derivatives and the *β*-isomers of 3-monoaroylated derivatives have also been clarified such as (2,7-dimethoxynaphthalen-1-yl)(2,4,6-trimethylphenyl)methanone (Muto *et al.*, 2011*a*) and (3,6-dimethoxynaphthalen-2-yl)(2,4,6-trimethylphenyl)methanone (Muto *et al.*, 2011*b*).

As a part of our continuing study on the molecular structures of these homologous molecules, the crystal structure of title compound, *peri*-aroylnaphthalene bearing three methyl groups, is discussed in this article.

The molecular structure of the title compound is displayed in Fig. 1. Two 2,4,6-trimethylphenyl groups are out of the plane of the naphthalene ring. The interplanar angle between the best planes of the two phenyl rings is 71.43 (7)°. On the other hand, the two interplanar angles between the best planes of the 2,4,6-trimethylphenyl rings and the naphthalene ring are 81.58 (5) and 84.92 (6)°, respectively. The torsion angles between the carbonyl groups and the naphthalene ring [C2—C1—C11—O1 = -104.68 (15)° and C10—C9—C18—O2 = 52.5 (2)°] are comparable with those between the carbonyl groups and 2,4,6-trimethylphenyl groups [O1—C11—C12—C17 = -141.08 (14)° and O2—C18—C19—C24 = -127.49 (14)°].

In addition, an intramolecular C—H···O interaction between a methyl group and carbonyl group is observed (C29—H29*c*···O2 = 2.33 Å; Fig. 2 and Table 1).

### Experimental

To a 10 ml flask, 1,8-bis(2,4,6-trimethylbenzoyl)-2-hydroxy-7-methoxynaphthalene (0.20 mmol, 0.093 g), dimethyl sulfate (0.40 mmol, 0.050 g), 2 *M* aqueous NaOH (0.162 g) and acetone (0.50 ml) were placed and stirred at 0°C for 1 h. The pale yellow precipitates were collected with suction filtration after removal of acetone from the solution under reduced pressure. The crude product was purified by recrystallization from hexane and CHCl_3_(3:1 *v*/*v*, yield 65%).

^1^H NMR δ (400 MHz, CDCl_3_); 2.23 (12*H*, s), 2.25 (6*H*, s), 3.45 (6*H*, s), 6.80 (4*H*, s), 7.16 (2*H*, d, *J* = 9.2 Hz), 7.92 (2*H*, d, *J* = 9.2 Hz) p.p.m..

^13^C NMR δ (75 MHz, CDCl_3_); 21.08, 21.69, 56.84, 112.64, 125.31, 125.70, 129.06, 129.60, 132.79, 137.66, 138.74, 139.18, 158.30, 198.80 p.p.m..

IR (KBr); 1666 (C=O), 1608, 1512, 1460 (Ar, naphthalene), 1271 (=C—O—C) cm^-1^.

HRMS (*m/z*); [*M* + H]^+^ Calcd for C_32_H_33_O_4_, 481.2379; found, 481.2386.

m.p. = 531.5–535.0 K.

### Refinement

All H atoms were found in a difference map and were subsequently refined as riding atoms, with C—H = 0.95 (aromatic) and 0.98 (methyl) Å, and with *U*_ĩso_(H) = 1.2 *U*_eq_(C).

### Crystal data

**Table:**

C_32_H_32_O_4_	*F*(000) = 1024
*M**_r_* = 480.58	*D*_x_ = 1.256 Mg m^−^^3^
Monoclinic, *P*2_1_/*n*	Cu *K*α radiation, λ = 1.54187 Å
Hall symbol: -P 2yn	Cell parameters from 38210 reflections
*a* = 7.71685 (14) Å	θ = 3.0–68.2°
*b* = 29.2344 (5) Å	µ = 0.65 mm^−^^1^
*c* = 11.5567 (2) Å	*T* = 193 K
β = 102.879 (1)°	Platelet, colorless
*V* = 2541.57 (8) Å^3^	0.60 × 0.40 × 0.10 mm
*Z* = 4	

### Data collection

**Table:**

Rigaku R-AXIS RAPID diffractometer	4657 independent reflections
Radiation source: rotaiting anode	4131 reflections with *I* > 2σ(*I*)
graphite	*R*_int_ = 0.054
Detector resolution: 10.000 pixels mm^-1^	θ_max_ = 68.2°, θ_min_ = 3.0°
ω scans	*h* = −9→9
Absorption correction: numerical (*NUMABS*; Higashi, 1999)	*k* = −35→35
*T*_min_ = 0.697, *T*_max_ = 0.938	*l* = −13→13
45450 measured reflections	

### Refinement

**Table:**

Refinement on *F*^2^	Secondary atom site location: difference Fourier map
Least-squares matrix: full	Hydrogen site location: inferred from neighbouring sites
*R*[*F*^2^ > 2σ(*F*^2^)] = 0.041	H-atom parameters constrained
*wR*(*F*^2^) = 0.121	*w* = 1/[σ^2^(*F*_o_^2^) + (0.0645*P*)^2^ + 0.6531*P*] where *P* = (*F*_o_^2^ + 2*F*_c_^2^)/3
*S* = 1.09	(Δ/σ)_max_ = 0.001
4657 reflections	Δρ_max_ = 0.26 e Å^−^^3^
334 parameters	Δρ_min_ = −0.26 e Å^−^^3^
0 restraints	Extinction correction: *SHELXL97* (Sheldrick, 2008), Fc^*^=kFc[1+0.001xFc^2^λ^3^/sin(2θ)]^-1/4^
Primary atom site location: structure-invariant direct methods	Extinction coefficient: 0.0065 (4)

### Special details

**Table:**

Geometry. All e.s.d.'s (except the e.s.d. in the dihedral angle between two l.s. planes) are estimated using the full covariance matrix. The cell e.s.d.'s are taken into account individually in the estimation of e.s.d.'s in distances, angles and torsion angles; correlations between e.s.d.'s in cell parameters are only used when they are defined by crystal symmetry. An approximate (isotropic) treatment of cell e.s.d.'s is used for estimating e.s.d.'s involving l.s. planes.
Refinement. Refinement of *F*^2^ against ALL reflections. The weighted *R*-factor *wR* and goodness of fit *S* are based on *F*^2^, conventional *R*-factors *R* are based on *F*, with *F* set to zero for negative *F*^2^. The threshold expression of *F*^2^ > σ(*F*^2^) is used only for calculating *R*-factors(gt) *etc*. and is not relevant to the choice of reflections for refinement. *R*-factors based on *F*^2^ are statistically about twice as large as those based on *F*, and *R*- factors based on ALL data will be even larger.

### Fractional atomic coordinates and isotropic or equivalent isotropic displacement parameters (Å^2^)

**Table:**

	*x*	*y*	*z*	*U*_iso_*/*U*_eq_	
O1	0.95452 (13)	0.10635 (4)	0.36709 (9)	0.0395 (3)	
O2	0.76176 (14)	0.18514 (4)	0.40208 (9)	0.0422 (3)	
O3	0.77613 (16)	0.00902 (3)	0.40754 (9)	0.0468 (3)	
O4	0.79237 (15)	0.22810 (4)	0.69327 (9)	0.0442 (3)	
C1	0.77923 (17)	0.08017 (4)	0.49609 (12)	0.0308 (3)	
C2	0.76256 (19)	0.03309 (5)	0.50610 (12)	0.0358 (3)	
C3	0.7396 (2)	0.01203 (5)	0.61097 (14)	0.0427 (4)	
H3	0.7273	−0.0202	0.6153	0.051*	
C4	0.7356 (2)	0.03912 (5)	0.70604 (14)	0.0438 (4)	
H4	0.7187	0.0254	0.7772	0.053*	
C5	0.75588 (19)	0.08698 (5)	0.70228 (13)	0.0381 (3)	
C6	0.7490 (2)	0.11343 (6)	0.80360 (14)	0.0465 (4)	
H6	0.7339	0.0984	0.8735	0.056*	
C7	0.7631 (2)	0.15955 (6)	0.80389 (13)	0.0454 (4)	
H7	0.7543	0.1767	0.8721	0.054*	
C8	0.79116 (19)	0.18170 (5)	0.70152 (13)	0.0364 (3)	
C9	0.80823 (17)	0.15785 (5)	0.60088 (12)	0.0311 (3)	
C10	0.78159 (17)	0.10901 (5)	0.59634 (12)	0.0313 (3)	
C11	0.80692 (18)	0.09463 (4)	0.37597 (12)	0.0307 (3)	
C12	0.65793 (18)	0.08897 (4)	0.26807 (12)	0.0321 (3)	
C13	0.6975 (2)	0.06856 (5)	0.16618 (13)	0.0387 (3)	
C14	0.5607 (2)	0.06311 (5)	0.06553 (13)	0.0480 (4)	
H14	0.5860	0.0482	−0.0019	0.058*	
C15	0.3893 (2)	0.07852 (6)	0.06005 (14)	0.0503 (4)	
C16	0.3537 (2)	0.09796 (5)	0.16122 (15)	0.0458 (4)	
H16	0.2369	0.1088	0.1589	0.055*	
C17	0.48211 (19)	0.10231 (5)	0.26663 (13)	0.0353 (3)	
C18	0.85140 (18)	0.18669 (4)	0.50261 (12)	0.0309 (3)	
C19	1.00640 (18)	0.21930 (4)	0.53521 (11)	0.0305 (3)	
C20	0.97596 (19)	0.26619 (5)	0.51092 (12)	0.0336 (3)	
C21	1.1143 (2)	0.29681 (5)	0.54902 (12)	0.0359 (3)	
H21	1.0922	0.3286	0.5369	0.043*	
C22	1.28434 (19)	0.28241 (5)	0.60434 (12)	0.0357 (3)	
C23	1.31281 (19)	0.23591 (5)	0.62309 (12)	0.0357 (3)	
H23	1.4293	0.2254	0.6579	0.043*	
C24	1.17612 (18)	0.20406 (5)	0.59248 (11)	0.0317 (3)	
C25	0.7598 (2)	−0.03941 (5)	0.40790 (15)	0.0455 (4)	
H25A	0.6384	−0.0477	0.4123	0.055*	
H25B	0.8440	−0.0520	0.4768	0.055*	
H25C	0.7858	−0.0519	0.3349	0.055*	
C26	0.8348 (3)	0.25455 (6)	0.79970 (17)	0.0618 (5)	
H26A	0.9325	0.2399	0.8564	0.074*	
H26B	0.7303	0.2566	0.8344	0.074*	
H26C	0.8709	0.2854	0.7814	0.074*	
C27	0.8788 (2)	0.05058 (6)	0.16233 (15)	0.0494 (4)	
H27A	0.9196	0.0296	0.2290	0.059*	
H27B	0.9623	0.0762	0.1681	0.059*	
H27C	0.8725	0.0343	0.0874	0.059*	
C28	0.2463 (3)	0.07440 (8)	−0.05266 (18)	0.0767 (7)	
H28A	0.2448	0.1022	−0.1003	0.092*	
H28B	0.1304	0.0705	−0.0324	0.092*	
H28C	0.2711	0.0479	−0.0982	0.092*	
C29	0.42200 (19)	0.12170 (5)	0.37219 (13)	0.0398 (3)	
H29A	0.4376	0.0986	0.4352	0.048*	
H29B	0.2963	0.1302	0.3485	0.048*	
H29C	0.4931	0.1488	0.4014	0.048*	
C30	1.2171 (2)	0.15426 (5)	0.62151 (14)	0.0394 (3)	
H30A	1.1781	0.1462	0.6940	0.047*	
H30B	1.3454	0.1491	0.6337	0.047*	
H30C	1.1543	0.1352	0.5557	0.047*	
C31	1.4320 (2)	0.31654 (5)	0.64281 (15)	0.0475 (4)	
H31A	1.4434	0.3239	0.7269	0.057*	
H31B	1.4048	0.3445	0.5953	0.057*	
H31C	1.5439	0.3035	0.6313	0.057*	
C32	0.7958 (2)	0.28456 (5)	0.45048 (15)	0.0442 (4)	
H32A	0.7051	0.2718	0.4886	0.053*	
H32B	0.7693	0.2758	0.3665	0.053*	
H32C	0.7960	0.3180	0.4571	0.053*	

### Atomic displacement parameters (Å^2^)

**Table:**

	*U*^11^	*U*^22^	*U*^33^	*U*^12^	*U*^13^	*U*^23^
O1	0.0345 (5)	0.0438 (6)	0.0404 (6)	−0.0039 (4)	0.0090 (4)	0.0047 (4)
O2	0.0482 (6)	0.0403 (6)	0.0350 (6)	−0.0085 (4)	0.0023 (5)	0.0027 (4)
O3	0.0756 (8)	0.0246 (5)	0.0426 (6)	0.0006 (5)	0.0183 (5)	0.0012 (4)
O4	0.0570 (7)	0.0351 (5)	0.0450 (6)	−0.0031 (5)	0.0210 (5)	−0.0096 (4)
C1	0.0301 (7)	0.0292 (7)	0.0325 (7)	−0.0001 (5)	0.0060 (5)	0.0036 (5)
C2	0.0391 (8)	0.0315 (7)	0.0362 (7)	0.0001 (6)	0.0072 (6)	0.0024 (6)
C3	0.0519 (9)	0.0318 (7)	0.0440 (8)	−0.0024 (6)	0.0100 (7)	0.0091 (6)
C4	0.0529 (9)	0.0416 (8)	0.0382 (8)	−0.0021 (7)	0.0130 (7)	0.0128 (6)
C5	0.0389 (8)	0.0414 (8)	0.0342 (7)	−0.0010 (6)	0.0088 (6)	0.0051 (6)
C6	0.0581 (10)	0.0502 (9)	0.0340 (8)	−0.0015 (7)	0.0162 (7)	0.0057 (6)
C7	0.0535 (9)	0.0515 (9)	0.0342 (8)	−0.0009 (7)	0.0161 (7)	−0.0054 (6)
C8	0.0353 (7)	0.0374 (7)	0.0372 (7)	−0.0014 (6)	0.0097 (6)	−0.0032 (6)
C9	0.0286 (7)	0.0321 (7)	0.0324 (7)	−0.0006 (5)	0.0062 (5)	0.0000 (5)
C10	0.0289 (7)	0.0330 (7)	0.0318 (7)	0.0000 (5)	0.0061 (5)	0.0031 (5)
C11	0.0344 (7)	0.0234 (6)	0.0349 (7)	0.0007 (5)	0.0092 (6)	0.0005 (5)
C12	0.0383 (7)	0.0258 (6)	0.0321 (7)	−0.0053 (5)	0.0076 (6)	0.0038 (5)
C13	0.0527 (9)	0.0300 (7)	0.0351 (7)	−0.0083 (6)	0.0130 (6)	0.0006 (6)
C14	0.0721 (12)	0.0388 (8)	0.0333 (8)	−0.0163 (8)	0.0120 (7)	−0.0025 (6)
C15	0.0599 (11)	0.0436 (9)	0.0397 (8)	−0.0170 (8)	−0.0053 (7)	0.0063 (7)
C16	0.0405 (8)	0.0410 (8)	0.0508 (9)	−0.0075 (6)	−0.0007 (7)	0.0079 (7)
C17	0.0364 (7)	0.0298 (7)	0.0386 (8)	−0.0059 (5)	0.0057 (6)	0.0059 (5)
C18	0.0335 (7)	0.0264 (6)	0.0334 (7)	0.0027 (5)	0.0090 (6)	−0.0021 (5)
C19	0.0354 (7)	0.0276 (6)	0.0299 (6)	−0.0009 (5)	0.0105 (5)	−0.0011 (5)
C20	0.0391 (8)	0.0301 (7)	0.0340 (7)	0.0015 (5)	0.0134 (6)	0.0001 (5)
C21	0.0471 (8)	0.0257 (6)	0.0383 (7)	−0.0007 (6)	0.0170 (6)	−0.0013 (5)
C22	0.0411 (8)	0.0336 (7)	0.0352 (7)	−0.0060 (6)	0.0147 (6)	−0.0068 (6)
C23	0.0345 (7)	0.0368 (7)	0.0359 (7)	−0.0008 (6)	0.0079 (6)	−0.0038 (6)
C24	0.0358 (7)	0.0301 (7)	0.0300 (7)	0.0002 (5)	0.0089 (5)	−0.0017 (5)
C25	0.0571 (10)	0.0264 (7)	0.0514 (9)	−0.0004 (6)	0.0084 (7)	0.0006 (6)
C26	0.0659 (12)	0.0492 (10)	0.0612 (11)	0.0019 (9)	−0.0051 (9)	−0.0211 (8)
C27	0.0612 (10)	0.0424 (9)	0.0512 (9)	−0.0043 (7)	0.0262 (8)	−0.0096 (7)
C28	0.0904 (16)	0.0686 (13)	0.0539 (11)	−0.0208 (11)	−0.0208 (10)	0.0034 (10)
C29	0.0332 (7)	0.0397 (8)	0.0472 (9)	0.0016 (6)	0.0105 (6)	0.0059 (6)
C30	0.0392 (8)	0.0314 (7)	0.0456 (8)	0.0026 (6)	0.0051 (6)	0.0006 (6)
C31	0.0489 (9)	0.0403 (8)	0.0551 (10)	−0.0114 (7)	0.0151 (7)	−0.0115 (7)
C32	0.0467 (9)	0.0338 (7)	0.0508 (9)	0.0070 (6)	0.0081 (7)	0.0027 (6)

### Geometric parameters (Å, °)

**Table:**

O1—C11	1.2155 (17)	C19—C24	1.4020 (19)
O2—C18	1.2133 (17)	C19—C20	1.4085 (18)
O3—C2	1.3624 (17)	C20—C21	1.388 (2)
O3—C25	1.4217 (17)	C20—C32	1.509 (2)
O4—C8	1.3600 (18)	C21—C22	1.390 (2)
O4—C26	1.4279 (19)	C21—H21	0.9500
C1—C2	1.3895 (19)	C22—C23	1.387 (2)
C1—C10	1.4297 (19)	C22—C31	1.505 (2)
C1—C11	1.5111 (18)	C23—C24	1.3923 (19)
C2—C3	1.405 (2)	C23—H23	0.9500
C3—C4	1.360 (2)	C24—C30	1.5115 (19)
C3—H3	0.9500	C25—H25A	0.9800
C4—C5	1.410 (2)	C25—H25B	0.9800
C4—H4	0.9500	C25—H25C	0.9800
C5—C6	1.414 (2)	C26—H26A	0.9800
C5—C10	1.4357 (19)	C26—H26B	0.9800
C6—C7	1.353 (2)	C26—H26C	0.9800
C6—H6	0.9500	C27—H27A	0.9800
C7—C8	1.407 (2)	C27—H27B	0.9800
C7—H7	0.9500	C27—H27C	0.9800
C8—C9	1.3873 (19)	C28—H28A	0.9800
C9—C10	1.4418 (19)	C28—H28B	0.9800
C9—C18	1.5100 (18)	C28—H28C	0.9800
C11—C12	1.5051 (19)	C29—H29A	0.9800
C12—C17	1.408 (2)	C29—H29B	0.9800
C12—C13	1.413 (2)	C29—H29C	0.9800
C13—C14	1.395 (2)	C30—H30A	0.9800
C13—C27	1.504 (2)	C30—H30B	0.9800
C14—C15	1.385 (3)	C30—H30C	0.9800
C14—H14	0.9500	C31—H31A	0.9800
C15—C16	1.382 (3)	C31—H31B	0.9800
C15—C28	1.513 (2)	C31—H31C	0.9800
C16—C17	1.394 (2)	C32—H32A	0.9800
C16—H16	0.9500	C32—H32B	0.9800
C17—C29	1.509 (2)	C32—H32C	0.9800
C18—C19	1.5102 (18)		
			
C2—O3—C25	119.24 (11)	C19—C20—C32	122.29 (13)
C8—O4—C26	118.92 (13)	C20—C21—C22	122.09 (13)
C2—C1—C10	120.10 (12)	C20—C21—H21	119.0
C2—C1—C11	112.84 (12)	C22—C21—H21	119.0
C10—C1—C11	126.90 (11)	C23—C22—C21	117.98 (13)
O3—C2—C1	114.59 (12)	C23—C22—C31	121.37 (14)
O3—C2—C3	122.87 (13)	C21—C22—C31	120.65 (13)
C1—C2—C3	122.51 (13)	C22—C23—C24	122.10 (13)
C4—C3—C2	118.13 (14)	C22—C23—H23	119.0
C4—C3—H3	120.9	C24—C23—H23	119.0
C2—C3—H3	120.9	C23—C24—C19	118.85 (12)
C3—C4—C5	122.03 (13)	C23—C24—C30	118.60 (12)
C3—C4—H4	119.0	C19—C24—C30	122.55 (12)
C5—C4—H4	119.0	O3—C25—H25A	109.5
C4—C5—C6	119.49 (13)	O3—C25—H25B	109.5
C4—C5—C10	120.59 (13)	H25A—C25—H25B	109.5
C6—C5—C10	119.92 (14)	O3—C25—H25C	109.5
C7—C6—C5	121.95 (14)	H25A—C25—H25C	109.5
C7—C6—H6	119.0	H25B—C25—H25C	109.5
C5—C6—H6	119.0	O4—C26—H26A	109.5
C6—C7—C8	118.95 (14)	O4—C26—H26B	109.5
C6—C7—H7	120.5	H26A—C26—H26B	109.5
C8—C7—H7	120.5	O4—C26—H26C	109.5
O4—C8—C9	116.04 (12)	H26A—C26—H26C	109.5
O4—C8—C7	121.52 (13)	H26B—C26—H26C	109.5
C9—C8—C7	122.33 (14)	C13—C27—H27A	109.5
C8—C9—C10	119.26 (12)	C13—C27—H27B	109.5
C8—C9—C18	115.35 (12)	H27A—C27—H27B	109.5
C10—C9—C18	125.38 (11)	C13—C27—H27C	109.5
C1—C10—C5	116.56 (12)	H27A—C27—H27C	109.5
C1—C10—C9	126.13 (12)	H27B—C27—H27C	109.5
C5—C10—C9	117.31 (12)	C15—C28—H28A	109.5
O1—C11—C12	121.21 (12)	C15—C28—H28B	109.5
O1—C11—C1	118.92 (12)	H28A—C28—H28B	109.5
C12—C11—C1	119.47 (11)	C15—C28—H28C	109.5
C17—C12—C13	119.60 (13)	H28A—C28—H28C	109.5
C17—C12—C11	122.37 (12)	H28B—C28—H28C	109.5
C13—C12—C11	118.02 (13)	C17—C29—H29A	109.5
C14—C13—C12	118.63 (15)	C17—C29—H29B	109.5
C14—C13—C27	118.13 (14)	H29A—C29—H29B	109.5
C12—C13—C27	123.19 (14)	C17—C29—H29C	109.5
C15—C14—C13	122.58 (15)	H29A—C29—H29C	109.5
C15—C14—H14	118.7	H29B—C29—H29C	109.5
C13—C14—H14	118.7	C24—C30—H30A	109.5
C16—C15—C14	117.59 (14)	C24—C30—H30B	109.5
C16—C15—C28	121.12 (18)	H30A—C30—H30B	109.5
C14—C15—C28	121.29 (17)	C24—C30—H30C	109.5
C15—C16—C17	122.69 (16)	H30A—C30—H30C	109.5
C15—C16—H16	118.7	H30B—C30—H30C	109.5
C17—C16—H16	118.7	C22—C31—H31A	109.5
C16—C17—C12	118.71 (14)	C22—C31—H31B	109.5
C16—C17—C29	116.99 (14)	H31A—C31—H31B	109.5
C12—C17—C29	124.30 (12)	C22—C31—H31C	109.5
O2—C18—C9	121.68 (12)	H31A—C31—H31C	109.5
O2—C18—C19	120.87 (12)	H31B—C31—H31C	109.5
C9—C18—C19	117.39 (11)	C20—C32—H32A	109.5
C24—C19—C20	120.01 (12)	C20—C32—H32B	109.5
C24—C19—C18	121.45 (12)	H32A—C32—H32B	109.5
C20—C19—C18	118.50 (12)	C20—C32—H32C	109.5
C21—C20—C19	118.83 (13)	H32A—C32—H32C	109.5
C21—C20—C32	118.81 (13)	H32B—C32—H32C	109.5
			
C25—O3—C2—C1	−179.58 (13)	C1—C11—C12—C13	−132.32 (13)
C25—O3—C2—C3	2.5 (2)	C17—C12—C13—C14	1.36 (19)
C10—C1—C2—O3	−175.05 (12)	C11—C12—C13—C14	−179.99 (12)
C11—C1—C2—O3	0.71 (17)	C17—C12—C13—C27	−176.01 (13)
C10—C1—C2—C3	2.9 (2)	C11—C12—C13—C27	2.63 (19)
C11—C1—C2—C3	178.64 (13)	C12—C13—C14—C15	2.6 (2)
O3—C2—C3—C4	177.08 (14)	C27—C13—C14—C15	−179.89 (14)
C1—C2—C3—C4	−0.7 (2)	C13—C14—C15—C16	−3.2 (2)
C2—C3—C4—C5	−0.8 (2)	C13—C14—C15—C28	176.61 (15)
C3—C4—C5—C6	179.44 (15)	C14—C15—C16—C17	−0.2 (2)
C3—C4—C5—C10	0.1 (2)	C28—C15—C16—C17	179.99 (15)
C4—C5—C6—C7	−178.31 (16)	C15—C16—C17—C12	4.0 (2)
C10—C5—C6—C7	1.1 (2)	C15—C16—C17—C29	−176.53 (14)
C5—C6—C7—C8	−2.0 (3)	C13—C12—C17—C16	−4.53 (19)
C26—O4—C8—C9	−159.31 (14)	C11—C12—C17—C16	176.88 (12)
C26—O4—C8—C7	24.5 (2)	C13—C12—C17—C29	176.08 (13)
C6—C7—C8—O4	174.71 (15)	C11—C12—C17—C29	−2.5 (2)
C6—C7—C8—C9	−1.3 (2)	C8—C9—C18—O2	−126.39 (14)
O4—C8—C9—C10	−170.79 (12)	C10—C9—C18—O2	52.54 (19)
C7—C8—C9—C10	5.4 (2)	C8—C9—C18—C19	50.95 (16)
O4—C8—C9—C18	8.21 (18)	C10—C9—C18—C19	−130.12 (13)
C7—C8—C9—C18	−175.61 (13)	O2—C18—C19—C24	−127.50 (14)
C2—C1—C10—C5	−3.44 (19)	C9—C18—C19—C24	55.14 (17)
C11—C1—C10—C5	−178.55 (12)	O2—C18—C19—C20	54.91 (18)
C2—C1—C10—C9	176.19 (13)	C9—C18—C19—C20	−122.46 (13)
C11—C1—C10—C9	1.1 (2)	C24—C19—C20—C21	−2.66 (19)
C4—C5—C10—C1	2.0 (2)	C18—C19—C20—C21	174.97 (12)
C6—C5—C10—C1	−177.33 (13)	C24—C19—C20—C32	−179.48 (13)
C4—C5—C10—C9	−177.62 (13)	C18—C19—C20—C32	−1.85 (19)
C6—C5—C10—C9	3.0 (2)	C19—C20—C21—C22	3.7 (2)
C8—C9—C10—C1	174.28 (13)	C32—C20—C21—C22	−179.37 (13)
C18—C9—C10—C1	−4.6 (2)	C20—C21—C22—C23	−1.2 (2)
C8—C9—C10—C5	−6.08 (19)	C20—C21—C22—C31	179.04 (13)
C18—C9—C10—C5	175.03 (12)	C21—C22—C23—C24	−2.5 (2)
C2—C1—C11—O1	−104.69 (15)	C31—C22—C23—C24	177.31 (13)
C10—C1—C11—O1	70.72 (18)	C22—C23—C24—C19	3.4 (2)
C2—C1—C11—C12	68.13 (16)	C22—C23—C24—C30	−177.05 (13)
C10—C1—C11—C12	−116.46 (15)	C20—C19—C24—C23	−0.79 (19)
O1—C11—C12—C17	−141.06 (14)	C18—C19—C24—C23	−178.34 (12)
C1—C11—C12—C17	46.28 (17)	C20—C19—C24—C30	179.71 (12)
O1—C11—C12—C13	40.34 (18)	C18—C19—C24—C30	2.15 (19)

### Hydrogen-bond geometry (Å, °)

**Table:**

*D*—H···*A*	*D*—H	H···*A*	*D*···*A*	*D*—H···*A*
C29—H29C···O2	0.98	2.33	3.1669 (19)	143
